# Deciphering the disease ecosystem of ischemic stroke via multi-omics and prospects for therapeutic strategies

**DOI:** 10.3389/fnins.2026.1740084

**Published:** 2026-02-17

**Authors:** Jingwen Zhang, Jiajie Niu, Jingwen Zhao, Linjing Wang, Tuozi Wang, Shuai Shi

**Affiliations:** 1 Heilongjiang University of Chinese Medicine, Graduate School, Harbin, China; 2 The Second Affiliated Hospital of Heilongjiang University of Chinese Medicine, Department of Acupuncture, Harbin, Heilongjiang, China; 3 The Second Affiliated Hospital of Heilongjiang University of Chinese Medicine, Department of Geriatrics, Harbin, Heilongjiang, China

**Keywords:** brain-peripheral immune axis, cellular identity collapse, disease ecosystem, ischemic cerebral infarction, neurovascular unit

## Abstract

Ischemic stroke (IS) remains a leading cause of disability due to the translational failure of single-target therapies, underscoring the limitations of the traditional neuron-centric view. This review proposes a paradigm shift by conceptualizing IS as a dynamically evolving “disease ecosystem.” We integrate multi-omics evidence to delineate five interconnected core features—cellular identity collapse, inflammatory-reparative imbalance, neurovascular unit disintegration, brain-peripheral immune miscommunication, and extracellular matrix scarring—that form a pathological logic axis driving disease progression. Based on this systemic understanding, we advance the novel therapeutic strategy of “ecosystem engineering,” which emphasizes temporally adaptive, spatially precise, and network-coordinated interventions. This framework aims to overcome current therapeutic bottlenecks and usher in a new era of precision neural repair.

## Graphical Abstract

**Figure:**
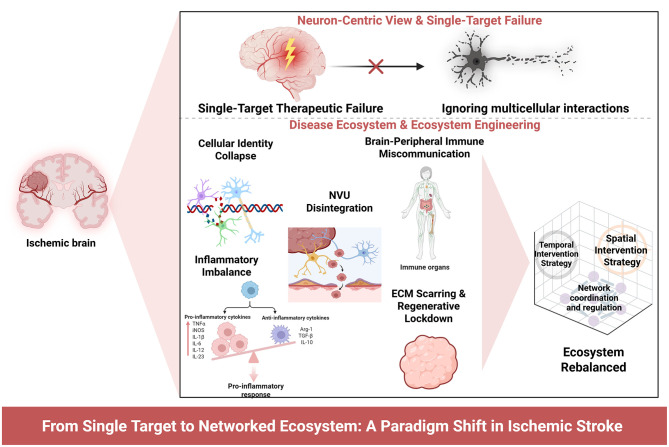
Decoding the ischemic stroke disease ecosystem: from systemic collapse to therapeutic remodeling. The graphical abstract conceptualizes the paradigm shift from a traditional neuron-centric view to a holistic “disease ecosystem” perspective in ischemic stroke. The left panel (Ecosystem Homeostasis) symbolizes the balanced multicellular community in a healthy brain. The central panel (Cascade Collapse) depicts the five interconnected core features (cellular identity collapse, inflammation-repair imbalance, NVU disintegration, brain-peripheral immune miscommunication, and ECM scarring) that drive ecosystem failure following ischemia. The right panel (Therapeutic Ecosystem Engineering) illustrates the proposed intervention strategy, guided by three core principles: temporally dynamic, spatially precise, and network-coordinated interventions, aimed at steering the damaged ecosystem from a pathological state toward functional recovery. “Graphical Abstract” created in BioRender. Hong, Zhi (https://BioRender.com/hj9yl80) is licensed under CC BY 4.0.Corresponding license: Publication License Jan-13-2026.pdf (Agreement number: JI298E4MNO).

## 1 Introduction

Ischemic stroke (IS) ranks among the leading causes of disability and mortality worldwide, accounting for approximately 70–80% of all stroke cases (GBD 2019 Stroke Collaborators, 2021). With population aging and the prevalence of cardiovascular risk factors, its socioeconomic burden continues to intensify. Although recent years have witnessed revolutionary advances in acute-phase treatment through reperfusion therapies (such as intravenous thrombolysis and endovascular thrombectomy), the narrow therapeutic time window and the occurrence of “ineffective reperfusion” in over half of cases profoundly reflect significant gaps in our understanding of stroke pathophysiology (Heiss, 2012). The overwhelming majority of patients inevitably suffer severe neurological deficits, compelling us to transcend existing paradigms and reexamine the complex process of ischemic brain injury at a more fundamental level.

Over the past decades, stroke research has witnessed numerous “neuroprotective agents” targeting the preservation of dying neurons fail in large Phase III clinical trials. Examples include NXY-059, Tirilazad, and minocycline, with their failures documented in top-tier journals like Stroke and the New England Journal of Medicine (Shuaib et al., 2007; The RANTTAS Investigators, 1996). This series of setbacks compelled the academic community to deeply reflect: our overly simplistic reduction of cerebral infarction to a linear event of “neuronal death due to hypoxia” may have severely underestimated its essence as a complex systemic disorder. Consequently, neuroprotective strategies targeting single pathways—such as excitotoxicity or oxidative stress—have failed to translate into successful clinical applications (Dirnagl, 2006), highlighting the limitations of traditional neuron-centric paradigms. In reality, the brain constitutes a highly collaborative ecosystem comprising neurons, glial cells, vascular cells, and immune cells. The infarct core and its surrounding penumbra are not a solo performance by neurons, but rather a “microenvironmental battlefield” where diverse cellular components dynamically interact through intricate molecular signaling networks. These cellular communications and interactions collectively determine the tissue's ultimate fate—whether it progresses to complete necrosis or initiates repair and remodeling (Iadecola and Anrather, 2011b).

Recent advances in systems biology have fundamentally transformed our understanding of stroke pathology. Breakthroughs like single-cell RNA sequencing (scRNA-seq), spatial transcriptomics, and advanced bioinformatics algorithms provide unprecedented microscopic resolution to decipher dynamic changes in ischemic brain regions at the cellular and subcellular levels. These techniques not only reveal astonishing heterogeneity within traditional cell classifications (e.g., microglia cannot be simply categorized by the M1/M2 dichotomy) but also precisely map the continuous dynamic landscape of cellular states as they evolve with disease progression (Bormann et al., 2024; Li et al., 2023b).

Building upon these revolutionary insights, we propose a new paradigm: the “ischemic stroke disease ecosystem.” This framework views the infarct core and penumbra as a biologically dynamic system governed by specific rules, whose outcome depends on the overall equilibrium of the interaction network among its components rather than the survival of individual cell types.

It is crucial to state at the outset that this ecosystem paradigm is primarily a conceptual and interpretative framework derived from integrating correlative multi-omics observations. The causal relationships and mechanistic hierarchies within this proposed ecosystem are largely hypothetical and require future validation. With this perspective in mind, we formally define the “ischemic stroke disease ecosystem” as: A spatially organized, hierarchically structured, and temporally evolving multicellular community within the ischemic brain region. This community encompasses neurons, glia, vascular cells, immune cells, and their extracellular matrix, which interact through biochemical, mechanical, and electrical signaling networks. The system's behavior emerges from non-linear interactions among its components, exhibits adaptive dynamics in response to injury and repair stimuli, and is constrained by energy-metabolic boundaries and physical microenvironmental constraints. Its pathological progression or resolution is determined by the collective stability and resilience of the entire interactive network, not by the state of any single component.

This ecosystem perspective differs fundamentally from related but distinct concepts. Unlike “pathological networks,” which primarily map molecular interactions, the disease ecosystem incorporates spatial architecture, temporal evolution, and multi-scale hierarchical organization—from subcellular compartments to whole-brain circuits. Compared to “systemic diseases” that emphasize whole-body dissemination, it focuses on local microenvironment dysregulation and its bidirectional crosstalk with systemic processes. While “microenvironmental dysregulation” describes local imbalance, the ecosystem framework emphasizes the self-organizing dynamics, emergent properties, and network-level resilience or collapse that arise from component interactions. Essentially, it shifts the analytical unit from discrete elements (molecules, cells) to their relational patterns and collective behaviors within a defined spatiotemporal context.

The “disease ecosystem” framework thereby provides four distinct analytical advantages over prior systems-level concepts. First, it explicitly incorporates spatial architecture and temporal dynamics as fundamental determinants of system behavior, not merely as descriptive context. Second, it treats emergent properties (e.g., network resilience or collapse) as central outcomes to be explained and therapeutically targeted. Third, it emphasizes multi-scale hierarchical organization, requiring the integration of subcellular, cellular, tissue, and inter-organ communication scales. Fourth, and most consequentially, it shifts the primary unit of analysis from discrete entities (a molecule, a cell type) to the pattern of relationships and collective behaviors within a defined spatiotemporal niche. Consequently, therapeutic strategies derived from this paradigm aim not to correct a single “broken part,” but to steer the phase transition of the entire relational network from a pathological attractor state toward a reparative basin of attraction.

With this conceptual foundation established, this review aims to systematically construct the theoretical framework for the disease ecosystem of IS. By integrating cutting-edge multi-omics evidence, we delve into the core characteristics and operational principles of this system, striving to translate mechanistic insights into innovative therapeutic strategies that may overcome clinical bottlenecks. This paradigm shift has the potential to provide a more comprehensive framework for understanding stroke pathophysiology and developing novel therapies, which may ultimately advance stroke treatment toward more effective approaches.

This systems-level perspective necessitates a shift from single-target pharmacology to interventions capable of remodeling the entire disease network. Interestingly, this conceptual shift finds resonance in holistic medical systems, such as traditional Chinese medicine, which emphasizes the interconnectedness of organ systems. The “brain-gut co-treatment” theory, for instance, posits a bidirectional axis where cerebral and enteric functions modulate each other—a concept increasingly supported by modern evidence implicating the gut microbiome and neuroimmune pathways in stroke outcomes (Carabotti et al., 2015; Cryan et al., 2018). This convergence suggests that therapeutic strategies capable of engaging multiple systems simultaneously, akin to ‘ecosystem engineering,' may be inherent to certain complex interventions. This theoretical extension provides a cross-disciplinary inspiration for stroke “ecosystem engineering”—that is, achieving functional reconstruction at the system level through holistic, dynamic, and networked interventions.

A critical methodological consideration underpinning this ecosystem framework is its foundation in correlative multi-omics observations. While single-cell and spatial transcriptomics provide unprecedented resolution of cellular states and potential interactions, they primarily generate hypothesis-forming correlative data. Throughout this review, we therefore explicitly distinguish between: (1) correlative multi-omics observations (e.g., co-expression patterns, inferred communication networks), (2) experimentally validated mechanisms (e.g., demonstrated through genetic perturbation or functional assays), and (3) hypothesis-driven interpretations (e.g., ecological analogies, network-level predictions). This tripartite distinction is maintained consistently across all five core features, ensuring that the strength of evidence matches the strength of interpretation.

## 2 Five core characteristics from the disease ecosystem perspective

The pathological process of IS transcends the paradigm of acute necrosis of localized neurons; it constitutes a dynamically evolving ecosystem characterized by the failure of multicellular community homeostasis. Drawing upon ecological theory, we conceptualize the infarct core and penumbra as a disturbed ecosystem where the breakdown of three fundamental ecological principles drives pathology: (1) the collapse of cellular niche specialization (functional identity loss), (2) the dysregulation of inter-population signaling networks (communication failure), and (3) the structural disintegration of the habitat architecture (spatial organization collapse). Through integration of multi-omics evidence, we distill five interdependent core pathological features that collectively embody this ecosystem breakdown. These features are not merely sequential pathological events but represent simultaneous, interacting dimensions of ecosystem collapse, each reflecting a distinct failure mode in the system's self-organizing capacity. They collectively form a dynamic pathological network where each feature both influences and is influenced by the others through positive and negative feedback loops. The following sections systematically analyze these features through an ecological lens, emphasizing how each represents a failure in maintaining ecosystem equilibrium.

### 2.1 Feature one: cell identity collapse and lineage plasticity

From an ecological perspective, cellular identity collapse represents the breakdown of functional niche specialization within the cellular community. In healthy brain tissue, distinct cell types occupy defined ecological niches, performing specialized functions that collectively maintain system homeostasis. Ischemic injury disrupts these niches, triggering dedifferentiation and loss of functional specialization—an ecological phenomenon analogous to the breakdown of species' trophic roles in a stressed ecosystem. Lineage plasticity further reflects the ecosystem's attempt to adapt to catastrophic environmental change, but often results in maladaptive “generalist” phenotypes that compromise system function. This feature exemplifies the principle that ecosystem stability depends on the maintenance of specialized functional roles within its constituent populations.

The normal maintenance of brain function relies on highly specialized gene expression programs that define distinct cellular identities. Ischemic injury disrupts this functional specialization through two interrelated phenomena: cellular identity collapse and lineage plasticity. Cellular identity collapse refers to the loss of cell-type-specific gene expression patterns, leading to functional impairment. Lineage plasticity describes the blurring of traditional cellular lineage boundaries, where cells acquire transcriptional signatures of alternative lineages. These phenomena, occurring earlier than previously recognized, have been hypothesized to be potential initiating or contributing factors in the broader ecosystem imbalance (Carabotti et al., 2015; Cryan et al., 2018; Iadecola and Anrather, 2011a; Moskowitz et al., 2010). However, their precise causative role relative to other microenvironmental changes remains to be definitively established.

The pathophysiological basis of the ischemic core is the abrupt collapse of energy metabolism. Neurons are particularly sensitive to this; insufficient energy supply disrupts neuronal ion homeostasis, triggering glutamate excitotoxicity, which in turn leads to extensive transcriptional reprogramming (Choi, 2020; Chi et al., 2018). Notably, a significant proportion of neurons do not immediately undergo apoptosis but instead enter a “stalled-like state” characterized by dedifferentiation and functional arrest at the transcriptomic level, exhibiting a phenotype markedly distinct from healthy neurons (Moskowitz et al., 2010). From an ecological perspective, this stalled state can be interpreted as a transition from a specialized functional role to a non-functional generalist phenotype—analogous to a common response of specialist species to rapid environmental change. While this state may temporarily enhance survival by reducing metabolic demands, it represents a loss of ecosystem function as these cells no longer contribute to network activity or support other cellular populations. However, whether this state represents true dedifferentiation and whether it possesses the potential to regain full functionality remains to be confirmed by further functional studies. This suggests that neuronal identity is not absolutely fixed but exhibits instability under stress. scRNA-seq technology provides systematic evidence for ecosystem-level disruption. For example, the study by (Zheng et al. 2022) performed scRNA-seq on 28,914 cells from the mouse ischemic cortex at 3 days post-stroke. Their analysis revealed that within ischemic regions, over 85% of neurons significantly downregulated identity-defining genes (SYN1: log2FC −2.3, *p* = 4.7e-15; MAP2: log2FC −1.8, *p* = 2.1e-09), while oligodendrocytes showed similar loss of myelin-related genes (MBP: log2FC −3.1, *p* = 8.9e-18). This coordinated downregulation across multiple cell types is correlated with an ecosystem-wide collapse of functional specialization, representing a systemic stress response that extends beyond isolated cellular dysfunction. This correlation does not establish causality, and whether identity collapse initiates or results from broader ecosystem failure remains an open experimental question.

More importantly, pseudo-time trajectory analysis of these data revealed that cells did not transition randomly but followed defined paths toward dedifferentiated states. Neurons clustered into three distinct trajectories: one toward apoptosis (12%), one toward a stalled state (68%), and one toward an aberrant proliferative state (20%). This bifurcation pattern illustrates how ecosystem perturbation creates alternative adaptive pathways, with the stalled state representing a potential “ecological refuge” that conserves energy but sacrifices function.

A critical caution is warranted when interpreting these transcriptomic patterns of plasticity. The trajectory analyses and transcriptional overlaps described here provide a rich, hypothesis-generating map of potential cellular state transitions. However, they fall short of proving functional lineage conversion (e.g., astrocyte-to-neuron). In ecological terms, observing that a species exhibits traits of another under stress does not mean it has changed its fundamental ecological niche. Analogously, transcriptional overlap may indicate functional broadening, identity crisis, or transcriptional noise under stress, rather than definitive fate switching. The field currently lacks consensus on whether adult glia genuinely undergo reprogramming into functional neurons after stroke *in vivo*, with conflicting results often attributable to methodological differences in lineage-tracing specificity and sensitivity. Thus, while multi-omics reveals fascinating correlative patterns, their causal interpretation regarding cellular reprogramming remains speculative and must be framed as such.

To quantify cellular interactions, re-analysis using CellChat (Jin et al., 2021) on this dataset shows that the strength of neuron-astrocyte communication decreased by 73% for synaptic support signals (e.g., NRG-ERBB pathway) but increased by 210% for stress-response signals (e.g., TGFB-TGFR pathway). This rewiring of interaction networks, inferred from computational analysis, illustrates a potential shift in how ecosystem components may transition from specialized cooperative relationships to generalized stress-response modes. These extensive transcriptional reprogramming events may be driven upstream by profound epigenetic dysregulation. Studies indicate that ischemia triggers extensive epigenetic landscape remodeling, including histone modifications (e.g., acetylation, methylation) and DNA methylation, thereby regulating the expression of cell identity genes (Patnala et al., 2017; Stanzione et al., 2020; Su et al., 2022; Brookes et al., 2018; Kim et al., 2007; Moskowitz et al., 2010; Choi, 2020; Zheng et al., 2022). These epigenetic mechanisms directly govern chromatin accessibility, systematically rewriting gene expression programs following energy crises. They serve as “programmatic switches” for cellular identity collapse and provide theoretical grounds for regulating cell fate through epigenetic intervention.

Reactive changes in glial cells similarly reflect cellular identity remodeling. Astrocytes undergo rapid reactive proliferation and hypertrophy after ischemia, shifting their gene expression profiles from supportive, homeostasis-maintaining states toward pro-inflammatory, scarring states (Sofroniew, 2020). This involves upregulation of gene sets associated with cell proliferation, migration, and inflammation (e.g., Gfap, Vim, Serpina3n) (Sofroniew, 2020; Qian et al., 2023; Zhang Q. et al., 2022; Bormann et al., 2024). Microglia dynamically transition between pro-inflammatory M1 and anti-inflammatory/reparative M2 phenotypes, potentially exacerbating secondary injury or supporting repair at different stages (Hammond et al., 2019). This dynamic shift reveals the high plasticity of glial cells post-ischemia and offers a potential window for immune and inflammatory interventions. More groundbreaking findings lie in the lineage plasticity exhibited by cells after ischemia. Pseudo-time trajectory analysis reveals that a substantial number of astrocytes and microglia depart from their resting state and enter a highly activated “reactive state” (Bormann et al., 2024; Li et al., 2023b; Scott et al., 2024). More groundbreaking findings lie in the lineage plasticity exhibited by cells after ischemia. Pseudo-time trajectory analysis reveals that a substantial number of astrocytes and microglia depart from their resting state and enter a highly activated “reactive state” (Bormann et al., 2024; Li et al., 2023b; Scott et al., 2024). More strikingly, scRNA-seq data indicate that a subset of astrocytes can exhibit transcriptomic signatures associated with differentiation toward neuron-like cells (e.g., expressing transcripts such as Dcx and NeuroD1) or oligodendrocyte precursor cells (e.g., expressing Pdgfra, Cspg4). Conversely, a fraction of oligodendrocyte precursor cells may also show transcriptional overlap with astrocytic marker genes (e.g., Aqp4, Aldh1l1) (Ma et al., 2022). It is crucial to emphasize that these observations are correlative and confined to the mRNA level; they do not constitute evidence of functional transdifferentiation *in vivo*. The presence of neuronal or glial lineage transcripts in astrocytes (or vice versa) may reflect stress-induced transcriptional dysregulation, cellular multipotency, or technical artifacts rather than bona fide fate conversion. Rigorous lineage-tracing studies combined with functional validation are required to determine whether these transcriptional shifts lead to stable changes in cellular identity or function. These transcriptional findings raise the hypothesis that traditional cellular lineage boundaries might be more fluid than previously recognized under severe pathological stress. However, it is essential to distinguish between transcriptional overlap and demonstrated cellular plasticity. The apparent “fluidity” observed at the mRNA level does not equate to proven functional transdifferentiation *in vivo*. Indeed, the field remains divided on whether ischemia induces true astrocyte-to-neuron or OPC-to-astrocyte conversion in the adult mammalian brain. Key studies reporting such conversion have been challenged by concerns over the specificity of lineage tracing, cell fusion events, and the lack of robust functional integration of purportedly new neurons into circuits. Therefore, while transcriptional overlap is an intriguing correlate of ecosystem stress, its contribution to repair or pathology remains an open question. The observed transcriptional blurring may reflect the ecosystem's chaotic attempt to respond to injury, with outcomes that are often mixed—potentially contributing to glial scar formation while also generating a microenvironment that is not conducive to authentic regeneration.

Collectively, the multi-omics evidence paints a compelling but correlative map of post-ischemic cellular identity collapse and transcriptional plasticity as a systemic stress response. However, a critical and unresolved question persists: does identity collapse act as a primary driver of the broader ecosystem failure, or is it itself a consequence of a prior, more fundamental microenvironmental breakdown? The logic chain we propose (energy metabolism collapse → cellular phenotype instability → lineage boundary blurring → microenvironment imbalance → functional repair impairment) serves as a testable hypothesis rather than an established causal sequence. Future studies must employ longitudinal single-cell epigenomics coupled with causal perturbation techniques—such as CRISPR-based lineage tracing or inducible ablation of specific transitional cell states—to validate the directionality and necessity of these relationships. Establishing this causality is paramount for identifying the most effective therapeutic nodes within the ecosystem. It is essential to distinguish between three levels of evidence in these observations: (1) Transcriptional overlap (co-expression of lineage-associated genes, as seen in scRNA-seq), (2) *In vitro* transdifferentiation (demonstrated in cultured cells under controlled conditions), and (3) *In vivo* functional reprogramming (validated by lineage tracing with functional integration). While the first is well-documented in omics data, and the second has been reported in some studies, the third remains controversial and lacks definitive evidence in adult stroke models. Therefore, throughout this section, we use ‘lineage plasticity' to refer primarily to the transcriptional phenomenon, with explicit caveats regarding its functional interpretation.

Vascular-associated cells also undergo significant identity alterations. Studies reveal that pathological stimuli such as ischemia in cerebrovascular diseases cause endothelial cells to lose blood–brain barrier (BBB) integrity, exhibiting gene expression patterns characteristic of endothelial-to-mesenchymal transdifferentiation (EndMT) (Xiao et al., 2026). Some cells develop phenotypes resembling fibroblasts or immune cells (Yang and Rosenberg, 2011). This phenotypic drift not only compromises the efficacy of angiogenesis but may also form abnormal microvascular networks, exacerbating pathological remodeling in the ischemic focus.

The reshaping of cellular identity exhibits marked spatial heterogeneity that defines distinct ecological zones within the disturbed ecosystem. The study by (Zhang et al. 2023) provides a quantitative spatial ecology analysis by integrating 10X Visium spatial transcriptomics (capturing 4,998 spatial spots across infarct core, penumbra, and contralateral hemisphere) with scRNA-seq data from 12,500 cells.

Their spatial deconvolution analysis revealed three distinct astrocyte ecological zones:

1. Core zone (0–200 μm from infarct border): Characterized by a “scar-forming” phenotype (Gfap+ Vim+ Serpina3n+). CellChat analysis showed these astrocytes primarily send inhibitory signals to neurons (Semaphorin-Plexin interactions increased 4.2-fold) and receive pro-inflammatory signals from infiltrating monocytes (CCL2-CCR2 interactions).
2. Penumbral zone (200–800 μm): Exhibits a “reparative” phenotype (BDNF+ NTF3+ S100a10+). These astrocytes show strong bidirectional communication with endothelial cells (Angpt1-Tie2 pathway activity increased 3.1-fold), suggesting active participation in vascular niche remodeling.
3. Distal zone (>800 μm): Shows an “alerted” phenotype (C3+ C4b+ Vim+), potentially representing an early warning system that broadcasts inflammatory signals to distant regions.

Quantitative spatial autocorrelation analysis (Moran's I = 0.67, *p* < 0.001) confirmed that these phenotypes are not randomly distributed but form spatially organized patches, analogous to vegetation zones around a disturbance in terrestrial ecosystems. The transition between zones follows a gradient pattern where the edge effect (interface between zones) shows the highest cellular plasticity, with 23% of cells expressing hybrid phenotypes.

This spatial patterning creates what ecologists term a “landscape of fear,” where cells in different zones experience distinct selective pressures. Cells in the core face extreme resource limitation (oxygen, glucose), favoring stress-tolerant generalists, while penumbral cells exist in a transitional zone where reparative phenotypes can compete more effectively. Earlier single-nucleus RNA sequencing studies have reported that, in certain contexts, a fraction of cells annotated as astrocytes can co-express a limited repertoire of transcripts that are also characteristic of neuronal lineages (Zhao et al., 2026). Similarly, under inflammatory conditions, some OPCs have been observed to transiently upregulate a subset of astrocyte-associated genes (Bormann et al., 2024). These findings underscore the transcriptional complexity revealed by omics technologies but highlight a critical gap between correlative signatures and functional fate change. As emphasized throughout this section, co-expression of lineage-associated transcripts is not synonymous with cellular reprogramming. The physiological relevance—whether these transcriptional shifts represent abortive differentiation attempts, adaptive stress responses, or mere epiphenomena—cannot be inferred from transcriptomics alone and awaits definitive testing through *in vivo* lineage-tracing and functional assays. Therefore, while cellular identity in ischemic environments exhibits transcriptional dynamism, its functional plasticity remains to be rigorously established. Future research must prioritize causal experiments that couple precise lineage tracing with assessments of cellular function and integration to distinguish correlation from causation in ecosystem remodeling.

Collectively, post-ischemic cellular identity collapse and lineage plasticity represent a systemic stress response across the entire ecosystem. Its fundamental logic chain can be summarized as: energy metabolism collapse → cellular phenotype instability → (potential) transcriptomic lineage blurring → microenvironment imbalance → functional repair impairment. It is important to reiterate that “lineage blurring” in this proposed chain refers primarily to the observed transcriptional phenomena and their postulated contribution to microenvironment dysregulation, not to a conclusively proven step of cellular transdifferentiation. This understanding not only explains why traditional target-based therapies have limited efficacy but also provides theoretical support for developing future integrated “reprogramming-stabilization-repair” strategies.

Ecosystem Insight from Multi-omics: The integrated data from Zheng and Zhang reveal that cellular identity collapse is not a random event but a spatially structured, system-level response. It follows an ecological rule: under severe perturbation, ecosystems conserve cellular ‘biomass' but sacrifice functional specialization, creating spatially segregated zones of generalized phenotypes. This loss of niche specialization fundamentally undermines the cooperative relationships that maintain the neurovascular unit, initiating a cascade of ecosystem failure.

### 2.2 Feature two: dynamic imbalance of inflammation-repair signals

In ecological terms, inflammation and repair represent competing signaling regimes that regulate population dynamics and tissue remodeling. The healthy brain maintains these signals in homeostatic balance, analogous to predator-prey dynamics that regulate population sizes. Ischemic injury disrupts this balance, creating a pathological state where pro-inflammatory signaling becomes the dominant regulatory regime, overwhelming repair mechanisms. This imbalance reflects a failure of negative feedback regulation—a core principle in ecosystem stability. The temporal and spatial heterogeneity of inflammation-repair signals further mirrors the patch dynamics observed in disturbed ecosystems, where local disturbances create mosaics of different successional stages.

Following IS, the inflammatory response participates in nearly every stage of the pathological process. Its complexity lies in its dual potential to cause secondary injury while also contributing to tissue repair and functional restoration. Research indicates that inflammation is not a homogeneous negative event but exhibits distinct spatiotemporal dynamics. Acute inflammation aids in clearing necrotic cells and debris, but excessive inflammatory responses or failure to transition to repair mode often exacerbate secondary damage and impede tissue functional recovery (Iadecola et al., 2020; Fu et al., 2015). Therefore, understanding and regulating the dynamic equilibrium of inflammation-repair signaling has become a crucial entry point for deciphering disease ecosystems.

Temporally, the inflammatory response undergoes a sequential process from acute attack to chronic repair. It can be broadly divided into three phases: acute, subacute, and chronic. During the acute phase (hours to days post-onset), the infarct zone rapidly releases damage-associated molecular patterns (DAMPs) such as high mobility group box 1 (HMGB1), ATP, and HSP70, activating microglia and peripheral immune cells. These signals trigger a cascade release of proinflammatory cytokines (e.g., IL-1β, TNF-α) through pathways like TLR and NLRP3 (Shichita et al., 2014; Denes et al., 2010). While this phase facilitates necrotic tissue clearance, it is often accompanied by BBB disruption and further neuronal apoptosis (Yang and Rosenberg, 2011). Evidence from animal models suggests that transitioning into the subacute phase (days to weeks), inflammation may shift toward reparative immunity. In these models, M2 microglia are upregulated, accompanied by increased anti-inflammatory factors like IL-10 and TGF-β, potentially promoting angiogenesis and neuroregeneration (Hu et al., 2015). However, if this transition is insufficient or impeded, inflammation persists in a pro-inflammatory state, leading to the chronic phase characterized by persistent neuroinflammation, glial scar formation, and impaired neural circuit reconstruction (Liddelow and Barres, 2017). Beyond temporal dynamics, multi-omics enables quantitative analysis of the inflammatory signaling network topology that governs ecosystem stability. Re-analysis of the scRNA-seq data from (Garcia-Bonilla et al. 2024) using NicheNet reveals that during the acute phase, the inflammatory network shifts from a decentralized, resilient structure to a hub-dominated, fragile one. Specifically:

a. Network centralization increases: In sham controls, the top 5% of signaling pathways accounted for 28% of total interaction strength. At 24 h post-stroke, this increased to 67%, indicating over-reliance on a few dominant pathways (predominantly TNF, IL-1, and CCL signaling).
b. Feedback loops are disrupted: Normally, anti-inflammatory pathways (IL-10, TGF-β) provide negative feedback on pro-inflammatory signals, creating homeostatic oscillations. Post-stroke, cross-inhibition between these pathway clusters decreased by 82%, creating sustained pro-inflammatory activation.
c. Cell-type specific roles emerge: Using differential communication analysis, microglia were identified as the primary signal amplifiers (sending 3.4 × more inflammatory signals than they receive), while astrocytes serve as signal integrators (receiving from 5.2 different cell types on average). Neutrophils function as positive feedback generators, creating self-reinforcing loops through IL-1β autocrine signaling.

This network analysis reveals that inflammation-repair imbalance is not merely about cytokine levels, but about the collapse of regulatory network architecture. The ecosystem loses its capacity for self-correction because the feedback mechanisms that normally dampen oscillations are disabled.

This dynamic and spatially heterogeneous perspective provides a plausible framework to understand why “static” anti-inflammatory therapies targeting single pro-inflammatory factors (e.g., TNF-α) have frequently failed in clinical trials. For instance, both Enlimomab and Natalizumab demonstrated no overall benefit in stroke clinical trials (Enlimomab Acute Stroke Trial Investigators, 2001; Elkind et al., 2020), underscoring the necessity for temporally precise interventions.

Strong evidence from both animal and human studies indicates that the inflammatory response exhibits marked regional specificity. The infarct core typically forms a potent pro-inflammatory environment due to cell death and extracellular matrix (ECM) degradation, where infiltrating neutrophils and monocytes amplify inflammation alongside activated microglia (Shi et al., 2019). In contrast, the penumbra exhibits complex dual effects: while inflammatory cells release free radicals and proteases that promote apoptosis, they also secrete repair-promoting molecules such as vascular endothelial growth factor (VEGF) and brain-derived neurotrophic factor (BDNF), accelerating vascular and neural regeneration (Spychala et al., 2017). Moreover, low-level inflammatory responses in distant regions or even the contralateral hemisphere may exert “systemic reprogramming” effects across the entire brain by modulating synaptic plasticity and neural network reorganization (Benakis et al., 2016).

In summary, the inflammation-repair process following IS is not unidirectional or linear but rather a dynamic network dependent on spatiotemporal context. Premature or excessive activation of inflammatory signals readily leads to secondary injury, while insufficient or delayed repair signals impede functional recovery (Chamorro et al., 2016). This clearly demonstrates that applying a “static” intervention strategy to a highly “dynamic” pathological process is futile, explaining why simple anti-inflammatory therapies targeting a single pro-inflammatory factor (such as TNF-α) struggle to achieve clinical success. A more promising future direction lies in spatiotemporally precise immune modulation, achieving an optimal balance between inflammation and repair across both temporal and spatial dimensions to maximize neural functional recovery.

Ecosystem Insight from Multi-omics: Re-analysis of scRNA-seq data using NicheNet 74 reveals that the inflammatory-repair imbalance is governed by a network-level rule: under stress, regulatory networks shift from a decentralized, resilient architecture to a fragile, hub-dominated one. The dramatic increase in network centralization (from 28% to 67% of interaction strength concentrated in the top 5% pathways) and the 82% decrease in cross-inhibitory feedback explain why the system loses its capacity for self-correction and becomes locked in a pro-inflammatory state.

### 2.3 Feature three: disintegration and abnormal remodeling of neurovascular units

The neurovascular unit constitutes the architectural foundation of the brain's cellular ecosystem, analogous to the physical habitat structure in natural ecosystems. Its components—endothelial cells, pericytes, astrocytes, and neurons—form interdependent relationships that maintain microenvironmental stability. Neurovascular units (NVUs) disintegration represents habitat fragmentation, disrupting the spatial connectivity necessary for cellular communication and metabolic exchange. Subsequent abnormal remodeling reflects maladaptive architectural changes that fail to restore functional connectivity, creating isolated cellular “patches” with limited exchange capacity. This feature highlights how ecosystem resilience depends on maintaining both structural integrity and functional connectivity of habitat architecture.

NVUs comprise diverse cellular and matrix components, including neurons, astrocytes, microglia, vascular endothelial cells, pericytes, and basement membranes. They serve as the core structure maintaining brain microenvironment homeostasis, BBB functional integrity, and neurovascular coupling responses (Iadecola, 2017; del Zoppo, 2010; Zlokovic, 2008). Within this unit, endothelial cells form the physical barrier of the BBB through tight junctions, while pericytes and the basement membrane jointly maintain microvascular stability. Astrocytic endfeet envelop the vascular wall and participate in regulating neurovascular coupling. Microglia, as resident immune cells in the brain, contribute to the dynamic equilibrium of the NVU by sensing microenvironmental changes and regulating inflammatory responses. This multicellular cooperative system ensures precise coordination of substance exchange, metabolic regulation, and neural activity within the brain. This highly integrated functional complex is the first to suffer damage following IS onset: abrupt energy metabolism imbalance triggers ATP supply insufficiency, disruption of ion gradients, and cascading excitotoxicity and oxidative stress. This directly leads to neuronal and glial cell apoptosis or necrosis, rapidly disrupting NVU homeostasis (Zlokovic, 2008; Du et al., 2022).

During the acute ischemic phase (hours to days), BBB dysfunction is a key manifestation of NVU disintegration. Downregulation of tight junction proteins in vascular endothelial cells (e.g., claudin-5, occludin, ZO-1/TJP1) weakens barrier integrity and increases permeability (Liu et al., 2012; Yang and Rosenberg, 2011). From an ecological perspective, BBB breakdown represents the collapse of a critical environmental filter—a structure that normally regulates species (cell) movement between compartments. This collapse allows invasive species (peripheral immune cells) to enter the brain ecosystem, where they disrupt local ecological balance by outcompeting native species for resources and altering the chemical environment. Concurrently, impaired structural support of the endothelium by astrocytic foot processes, coupled with pericytic detachment or dysfunction, diminishes capillary stability and exacerbates microcirculatory perfusion abnormalities (Zhou et al., 2022). This facilitates plasma protein and peripheral immune cell infiltration into the brain parenchyma, amplifying local inflammatory responses and leading to edema and secondary neuronal injury (Abbott et al., 2010). Recent single-cell and spatial transcriptomics studies further reveal pronounced BBB heterogeneity across brain regions during acute stroke, exacerbating regional differences in ischemic susceptibility and repair (Munji et al., 2019).

During the subacute and repair phases (days to weeks), the body attempts to restore NVU structure through vascular and cellular remodeling. Endothelial cells and pericytes proliferate, inducing neovascularization. However, in the ischemic-inflammatory microenvironment, these new vessels often exhibit structural and functional abnormalities: irregular lumens, disorganized endothelial alignment, significant leakage, and inadequate hemodynamic efficiency (Wang et al., 2021). Signaling pathways essential for vascular maturation and stabilization—such as ANGPT1/TIE2 and PDGF-B/PDGFRβ–remain relatively deficient, further limiting perfusion improvement (Armulik et al., 2011). Concurrently, reactive astrocytes proliferate and form glial scars, which isolate injury and contain inflammatory spread while simultaneously hindering axonal regeneration and network reconstruction by secreting inhibitory ECM components (e.g., CSPGs) (Sofroniew, 2014). Recent reviews indicate that astrocytic reactivity may exert a “double-edged sword” effect during stroke recovery: serving both as a barrier and a regulator of repair (Verkhratsky et al., 2023).

At the molecular level, multi-omics and single-cell transcriptomics studies reveal extensive remodeling of NVU-related signaling pathways: dynamic alterations in VEGF, Notch, Angiopoietin/Tie2, and ECM-receptor interactions simultaneously promote angiogenesis while potentially inducing vascular instability (Wu et al., 2023). Furthermore, endothelial cells undergo partial or reversible EndMT in ischemic inflammatory environments, characterized by decreased endothelial-specific markers and acquisition of fibroblast/mesenchymal-like phenotypes. This transformation leads to increased vascular wall stiffness and permeability, thereby impairing vascular function and tissue repair (Simons, 2023). Microglia and infiltrating macrophages dynamically switch between pro-inflammatory (M1) and reparative (M2) phenotypes. The cytokines, matrix metalloproteinases (MMPs), and reactive oxygen species they secrete may either promote angiogenesis and debris clearance or exacerbate matrix degradation and vascular destruction, exerting bidirectional effects in NVU remodeling (Lyu et al., 2021).

To quantify the disintegration of NVU cellular cooperation, we re-analyzed spatial transcriptomics data from (Han et al. 2024) using a novel cellular interaction entropy metric. This metric measures the predictability of cell-cell interactions within spatial neighborhoods (lower entropy = more structured interactions). In sham brains, NVU neighborhoods showed low interaction entropy (H = 1.23 ± 0.18 bits), reflecting highly organized, predictable cellular partnerships. At 7 days post-stroke, entropy increased dramatically in the core (H = 3.87 ± 0.42 bits) and penumbra (H = 2.56 ± 0.31 bits), indicating randomization of cellular interactions.

More importantly, the interaction specificity—the tendency for particular cell types to interact with specific partners—decreased by 78% for neuron-astrocyte interactions and 64% for endothelial-pericyte interactions. According to this computational metric, the loss of interaction specificity can be viewed through an ecological lens as analogous to generalist species replacing specialists, which would be predicted to reduce overall ecosystem efficiency.

Spatial cross-correlation analysis further revealed that while individual NVU components might be physically present, their spatial coordination is disrupted. The normal tight spatial correlation between neuronal activity markers (c-Fos) and vascular markers (CD31) (*r* = 0.89 in sham) dropped to *r* = 0.21 in the penumbra, indicating functional uncoupling even when structural components remain adjacent.

Spatial heterogeneity is a key feature of abnormal NVU remodeling: while extensive neovascularization is observed in the infarct core, these vessels exhibit fragile structures and severe leakage; vessels in the penumbra remain relatively stable but show limited regenerative capacity; microvessels distant from the lesion demonstrate moderate dilation and network reorganization, potentially contributing to remote plasticity and compensatory repair (Krupinski et al., 1994). This macroscopic spatial heterogeneity has been precisely elucidated at the molecular level. Single-cell and spatial transcriptomics studies directly reveal distinctly different gene expression profiles in endothelial cells, pericytes, and astrocytes across distinct brain regions (e.g., infarct core, penumbra, and contralateral hemisphere), driving region-specific remodeling patterns (Han et al., 2024). These findings provide crucial evidence for understanding the molecular basis of NVU remodeling. Furthermore, advanced imaging data confirms that this vascular-cellular remodeling is not an isolated local event but involves a systemic response at the whole-brain network level (Yu et al., 2024). This perspective linking local NVU events to systemic pathological processes lies at the core of the “disease ecosystem” paradigm, suggesting that successful NVU repair strategies must address both local microenvironment regulation and systemic factor management.

Overall, the evolution of the NVU after ischemia can be simplified into three consecutive yet overlapping phases: acute injury → structural disintegration → pathological remodeling (or incomplete repair). Imbalance in any of these stages can lead to impaired functional recovery, indicating that single-target interventions often yield unsatisfactory outcomes. More promising future strategies should focus on “holistic protection and coordinated remodeling of the NVU,” namely, simultaneously promoting vascular stabilization, BBB repair, inflammatory regulation, and neural network plasticity within precise time windows to achieve true functional recovery (Xing et al., 2012; Alkayed and Cipolla, 2023).

Ecosystem Insight from Multi-omics: Spatial transcriptomics analysis using cellular interaction entropy metrics 65 quantifies a key principle of habitat disintegration: NVU breakdown is characterized not by the loss of individual components, but by the randomization of their structured interactions. The increase in interaction entropy and the 78% decrease in neuron-astrocyte interaction specificity represent the ecological equivalent of specialist species being replaced by generalists, drastically reducing the efficiency and functional output of the entire unit.

### 2.4 Feature four: malfunctioning communication between the brain and peripheral immune systems

This feature represents the breakdown of cross-system communication—a critical aspect of ecosystem networks. In ecological terms, the brain and peripheral immune system constitute two interconnected but distinct ecosystems that normally exchange regulatory signals. Stroke-induced miscommunication creates a pathological “cross-system cascade” where disturbances in one system amplify dysfunction in the other. The initial immune hypermobilization reflects an overcompensatory response to perceived systemic threat, while subsequent immunosuppression represents system exhaustion—a pattern analogous to boom-bust cycles in overexploited ecosystems. This feature illustrates how ecosystem dysfunction often propagates across system boundaries through maladaptive signaling, creating a positive feedback loop of escalating damage.

IS is not confined to a localized event within brain tissue but represents a systemic pathological process affecting the entire body. Increasing evidence indicates that the brain and peripheral immune systems form a tightly interconnected central-peripheral immune axis post-stroke. Systemic inflammation and immune reprogramming significantly influence acute injury progression, infectious complications, and long-term neurological outcomes (Simats and Liesz, 2022; Zhang Z. et al., 2022). Under physiological conditions, this axis coordinates the clearance of necrotic debris and initiates repair programs. However, ischemic events disrupt this finely tuned communication network, causing severe spatiotemporal misalignment, signal amplification dysregulation, and misdirected responses—termed “miscommunication”—which drive secondary injury and ultimately lead to repair failure.

Acute phase (hours to days) immune overmobilization: Massive cellular necrosis within the ischemic focus releases damage-associated molecular patterns (DAMPs, e.g., HMGB1, ATP, nucleic acid fragments) and proinflammatory cytokines (IL-1β, TNF-α, IL-6). These endogenous alarm signals breach the compromised blood-brain barrier (BBB) into the peripheral circulation, stimulating immune organs like the spleen and bone marrow. This triggers rapid peripheral leukocyte mobilization and systemic inflammatory cascades (Simats and Liesz, 2022; Gao et al., 2023). Studies utilizing bulk transcriptomic deconvolution (e.g., CIBERSORTx) and peripheral-brain integrated single-cell analysis reveal a “wave-like” immunocellular mobilization dynamic in the periphery post-stroke: early-stage recruitment of neutrophils (including Ly6G^+^/S100A9^+^ populations) and CCR2^+^ monocytes occurs en masse, rapidly entering the circulation and infiltrating the brain parenchyma through the compromised BBB; followed by compensatory upregulation of regulatory lymphocytes and myeloid inhibitory phenotypes (Liu et al., 2022; Xu et al., 2022; Garcia-Bonilla et al., 2024). Liu R et al. quantified the dynamic proportional changes of distinct immune cell subpopulations during this mobilization process by deconvoluting patient peripheral blood transcriptomes using the CIBERSORTx algorithm (Liu et al., 2022); Meanwhile, Garcia-Bonilla L et al. employed scRNA-seq on brain-blood paired samples to directly capture the sequential migration trajectories of specific immune cells (e.g., pro-inflammatory monocytes) from the peripheral circulation to the cerebral infarction site, providing direct evidence for “wave-like” infiltration (Garcia-Bonilla et al., 2024). These recruited neutrophils and monocytes subsequently release proteases, matrix metalloproteinases (MMPs), and reactive oxygen species (ROS), forming a positive feedback inflammatory loop that exacerbates infarct volume and impairs marginal zone function (Simats and Liesz, 2022; Xu et al., 2022). Furthermore, activated CD4?/CD8? T cells secrete proinflammatory factors like IFN-γ, driving microglia/macrophages toward a proinflammatory (M1) phenotype. Some T cells may even recognize self-antigens exposed or modified during ischemia, triggering delayed autoimmune-like reactions that prolong inflammation and impede repair (Endres et al., 2022; Faura et al., 2021).

Subacute phase (days to weeks): Immunosuppression and dysregulation. Prolonged immune communication disruption carries severe consequences. Following acute mobilization, the peripheral immune system often enters a state of relative suppression or reprogramming known as stroke-induced immunodepression (SIID), characterized by splenic atrophy, peripheral lymphopenia, and increased proportions of immunosuppressive cells (e.g., regulatory T cells, myeloid suppressor cells) (Faura et al., 2021; Westendorp et al., 2022). SIID reduces excessive systemic inflammation but significantly increases the risk of hospital-acquired and community-acquired infections (e.g., pulmonary infections). Secondary infections can then exacerbate brain injury in a feedback loop, creating a vicious cycle (Westendorp et al., 2022; Vaghi et al., 2024). Furthermore, during the subacute phase, severe mismatches in the distribution and activation states of monocytes/macrophages between the brain and peripheral tissues occur. This leads to asynchrony in inflammatory-repair signaling between the infarct core and penumbra, thereby impeding neuroprotection and regeneration processes within the penumbra (Zhang Z. et al., 2022; Bormann et al., 2024).

Spatial and temporal heterogeneity: Multimodal imaging and single-cell/spatial transcriptomics studies reveal that the brain's immune landscape and peripheral immune responses exhibit high spatial and temporal heterogeneity—the infarct core often remains in a persistently pro-inflammatory state, while the penumbra may show more balanced inflammatory-repair signals. Distal or contralateral hemispheres may exhibit low-grade inflammation or compensatory immune remodeling. The cellular basis of this heterogeneity stems from the diverse states of glial cells (e.g., microglia/macrophages) across different brain regions (Bormann et al., 2024). Its microenvironmental foundation arises from the intrinsic regional heterogeneity of cerebral vasculature and the BBB itself, along with their differential remodeling following ischemia (Pfau et al., 2024). When peripheral immune responses fail to switch from proinflammatory to reparative phenotypes within critical time windows, this heterogeneity leads to persistent core expansion, failure of penumbral rescue, and restricted whole-brain functional plasticity.

In summary, “brain-peripheral immune miscommunication” in stroke can be characterized by: mismatched local injury signals with peripheral immune responses, temporal misalignment between acute pro-inflammatory and subsequent immunosuppressive phases, and spatially heterogeneous inflammation-repair signal coordination. This “pendulum-like” response—shifting from early excessive inflammation to subsequent immunosuppression—reveals the complex temporal dynamics of stroke immunopathology. It not only amplifies intracerebral injury but also indirectly impedes repair by increasing systemic infection risk. Therefore, this phenomenon strongly suggests that future stroke therapies may require a “brain-periphery dual intervention” strategy. For example, during the acute phase, targeting the spleen or specific chemokines could limit the disordered infiltration of harmful leukocytes. Simultaneously, during the subacute phase, preventing or treating SIID-related infections could restore immune homeostasis (Simats and Liesz, 2022; Endres et al., 2022).

Ecosystem Insight from Multi-omics: Integrated brain-blood scRNA-seq studies 74 uncover a systemic communication rule: cross-system signaling during crisis follows a maladaptive, ‘boom-bust' dynamic. The early ‘wave-like' hypermobilization of peripheral immune cells, followed by compensatory immunosuppression (SIID), creates a temporal mismatch that amplifies injury and impedes repair. This reflects a failure of homeostatic feedback across ecosystem boundaries, where an initial overcompensation leads to systemic exhaustion.

### 2.5 Feature five: scarring of the extracellular matrix and the physicochemical barrier

ECM scarring represents the terminal consolidation of ecosystem dysfunction into a persistent inhibitory state. Ecologically, this process is analogous to the formation of “alternative stable states” in degraded ecosystems—persistent, low-functioning configurations that resist recovery. The scar creates both physical and chemical barriers that prevent recolonization by functional cell types and limit resource exchange, effectively locking the ecosystem into a pathological attractor state. This feature embodies the principle that ecosystems can become trapped in dysfunctional configurations through positive feedback mechanisms, requiring external intervention to overcome hysteresis and restore functional states.

The ECM serves as the “soil” and scaffold of the disease ecosystem, playing a double-edged role in pathological remodeling following ischemic stroke. It not only provides structural anchoring and mechanical support for cells but also acts as a key signaling source regulating cellular behavior. However, ischemia-triggered ECM remodeling exhibits a marked tendency toward pathological scarring, ultimately forming a regenerative barrier with both physical isolation and chemical inhibition properties. This barrier thus becomes a core pathological factor limiting neurovascular repair (Silver and Miller, 2004; Sofroniew, 2015).

During the acute phase, ischemia and reperfusion injury induce significant upregulation of matrix metalloproteinases (MMPs) such as MMP-2 and MMP-9, which rapidly degrade basement membrane collagen and tight junction proteins, directly compromising BBB structural integrity (Rosenberg, 2002). While this process facilitates clearance of necrotic cell debris, the resulting excessive degradation dramatically increases vascular permeability, triggering cerebral edema and inflammatory cell infiltration, thereby significantly exacerbating secondary injury (Yang and Rosenberg, 2011). Studies in MMP-9-deficient mouse models show that these animals exhibit markedly reduced post-stroke cerebral edema and hemorrhagic areas, suggesting their potential role in acute barrier disruption (Asahi et al., 2000; Romanic et al., 1998). Transition to Subacute and Repair Phases: Suppression of Scar Formation: As the pathological process progresses, reactive glial cells (astrocytes and oligodendrocyte precursor cells) proliferate rapidly and secrete large amounts of inhibitory molecules, particularly chondroitin sulfate proteoglycans (CSPGs, such as ACAN, VCAN, BCAN), collectively forming a glial scar (Bradbury et al., 2002; Cregg et al., 2014). CSPGs bind to neuronal surface receptors (PTPσ, LAR, NgR1), activating downstream RhoA/ROCK signaling pathways that powerfully inhibit axonal regeneration and synaptic plasticity (Sami et al., 2020; Miller and Hsieh-Wilson, 2015; Hu et al., 2022). Concurrently, glial scar formation creates direct physical barriers to the extension and maturation of neovascularization, limiting effective vascular network reconstruction (Burda and Sofroniew, 2014).

Beyond its chemical inhibitory effects, the physical properties of the ECM scar—specifically alterations in the mechanical microenvironment—profoundly influence cellular behavior. ECM remodeling exhibits a marked spatial gradient distribution within the brain. The infarct core represents the most severely scarred region, forming a nearly insurmountable physicochemical barrier. The ischemic penumbra exists in a “tug-of-war” state, harboring both regenerative potential and being permeated by inhibitory molecules from the core scar zone. Its ECM exhibits moderate inhibition, making it a critical target region where intervention strategies may reverse the outcome (Wanner et al., 2013). Notably, in brain areas distant from the lesion, the ECM composition remains relatively stable, potentially providing the necessary molecular foundation for compensatory neuroplasticity. This spatial heterogeneity indicates that the ECM scar exerts a global influence, remotely regulating the brain's functional remodeling process by altering its mechanical environment and molecular signaling networks. Thus, the ECM functions not merely as a passive barrier but as an active signaling platform, whose physicochemical properties collectively shape a microenvironment unfavorable for regeneration.

Given the core inhibitory role of ECM scarring, targeting its key components has emerged as a highly promising therapeutic approach. Preclinical studies have demonstrated that intracranial injection of chondroitin-acetyltransferase ABC (ChABC) to degrade the glycosaminoglycan side chains of CSPGs effectively attenuates their inhibitory effects, significantly promoting axonal regeneration and functional recovery (Bradbury et al., 2002). An alternative strategy bypasses challenging enzyme therapies by employing specific small-molecule peptides to competitively block CSPG binding to their receptor PTPσ, yielding similarly positive effects on neuroregeneration in animal models (Lang et al., 2015). This offers a concrete translational pathway for “unsealing” the ecosystem during the chronic phase.

In summary, post-ischemic ECM scarring represents the culmination of multiple pathological processes: cellular identity transformation, inflammation-repair imbalance, and NVU disintegration. It functions like a “barrier wall” erected at the injury site, simultaneously restricting harmful diffusion and impeding internal repair. Therefore, successful future repair strategies must incorporate the dimension of precise temporal regulation of the ECM. This entails appropriately controlling MMP-mediated degradation during the acute phase and skillfully mitigating CSPG inhibition during the repair phase. Such an approach dismantles this physicochemical barrier, paving the way for the regeneration of nerves and blood vessels. Collectively, these five core features form an interdependent network of ecosystem dysfunction, rather than a linear sequence of pathological events. Their relationships can be conceptualized as a dynamic system where: (1) Cellular identity collapse undermines the functional specialization necessary for NVU integrity; (2) NVU disintegration facilitates immune cell invasion and exacerbates inflammation-repair imbalance; (3) Dysregulated inflammation drives ECM remodeling toward scarring; (4) ECM scarring creates feedback that reinforces cellular identity collapse by restricting phenotypic plasticity; (5) Brain-peripheral immune miscommunication amplifies all other features by disrupting systemic regulation. This network perspective explains why interventions targeting single features often fail: they address symptoms without altering the underlying interaction network that generates and sustains the pathological ecosystem state.

Ecosystem Insight from Multi-omics: The spatial gradient of ECM remodeling reveals a fundamental principle of pathological succession: the ecosystem consolidates dysfunction into an ‘alternative stable state'—the scar. This state is maintained by positive feedback (e.g., inhibitory CSPGs reinforcing glial reactivity) and creates a dual physical-chemical barrier that actively prevents recolonization by functional cell types, effectively locking the tissue in a low-functioning, resilient pathological attractor.

## 3 Discussion and outlook: advancing toward an ecosystem engineering era in stroke treatment

**Figure 1:**
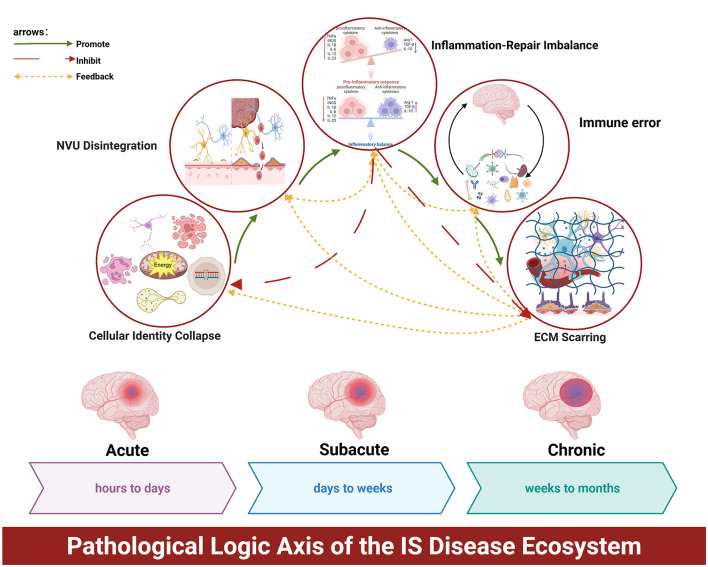
Pathological logic axis of the ischemic stroke disease ecosystem. This schematic integrates the five core pathological features into a dynamic causal network, interpreted through ecological analogies. The axis originates from an energy crisis, driving cellular identity collapse (ecological analogy: loss of functional niche specialization). This destabilizes the neurovascular unit (NVU), leading to its disintegration and blood-brain barrier (BBB) disruption (habitat fragmentation). BBB failure unleashes a cascade of inflammatory-repair imbalance (dysregulated population dynamics) and amplifies brain-peripheral immune miscommunication (cross-system cascade effects). These maladaptive processes culminate in extracellular matrix (ECM) scarring (formation of an alternative stable state), which establishes a persistent physicochemical barrier to regeneration. Solid arrows represent primary causal or temporal drivers, while dashed arrows denote critical feedback loops that reinforce the pathological ecosystem state. The model emphasizes the non-linear, interconnected nature of ecosystem failure over a simple linear sequence.This figure was created in BioRender. Hong, Zhi (https://BioRender.com/nggbubn) is licensed under CC BY 4.0.Corresponding license: Publication License Jan-13-2026 (1).pdf (Agreement number: SIZ98FD481).

It is important to reiterate that the “disease ecosystem” framework operates at three distinct but interacting levels of analysis: (1) Descriptive/Correlative (multi-omics mapping of cellular states and interactions), (2) Mechanistic/Experimental (validated causal relationships through perturbation studies), and (3) Conceptual/Interpretive (ecological analogies and systems principles). Throughout this discussion, we explicitly maintain this tri-level distinction to ensure that hypotheses derived from correlative observations are clearly framed as testable predictions rather than facts. Its five core characteristics—detailed below—are not isolated pathological events but interdependent variables within a dynamical system. Each characteristic influences and is influenced by others through feedback loops and cross-scale interactions, creating the non-linear progression patterns observed in stroke. This systems view explains why interventions targeting single characteristics often fail: they address system outputs without modifying the underlying interaction rules that generate those outputs. Through the layered analysis presented above, a comprehensive panorama of the IS “disease ecosystem”—characterized by intricate multicellular and multimolecular collaboration—has emerged (Jiang X. et al., 2018; Wang et al., 2021; Dzyubenko et al., 2018). The five core characteristics we have distilled—collapse of cellular identity and lineage plasticity, dynamic imbalance of inflammatory-reparative signaling, disintegration and abnormal remodeling of the neurovascular unit (NVU), miscommunication between the brain and peripheral immune systems, and scarring of the extracellular matrix (ECM)—are by no means an isolated list of pathologies (Wang et al., 2021; Wu et al., 2022; Wynn and Vannella, 2016; Li et al., 2023a). Together, they weave a highly integrated, causally interlinked dynamic pathological network (Figure 1) (Wang et al., 2021; Candelario-Jalil et al., 2022). Understanding the intrinsic operational principles and driving logic of this network holds the key to bridging the current knowledge gap and unlocking next-generation therapeutic strategies (Wu et al., 2022; Jayaraj et al., 2019). The core innovation of this review lies in systematically proposing the new paradigm of the “ischemic stroke disease ecosystem” and constructing a “pathological logic axis” composed of these five core features. This framework successfully integrates seemingly discrete pathological processes into a coherent, dynamic causal network, transcending traditional neuron-centric paradigms. Building upon this, we further propose the therapeutic philosophy of “Ecosystem Engineering.” Its core principles—temporal-dynamic intervention, spatially-specific navigation, and network-coordinated regulation—provide a clear conceptual framework and actionable roadmap for future therapeutic development. This framework offers both an explanation for past therapeutic challenges and a potential roadmap for developing next-generation stroke treatments.

### 3.1 Pathological logic axis of ecosystem succession

To more systematically understand the complex interactions within the disease ecosystem, we hereby propose an integrated model of the IS Pathological Logic Axis (Figure 1). This model not only reveals the intrinsic connections among the five core pathological features but, more importantly, elucidates their interlinked causal driving relationships throughout the disease process, forming a complete pathological logic chain from acute injury to chronic sequelae.

#### 3.1.1 Sequential initiation: energy crisis-triggered cellular identity collapse and NVU structural disintegration

The initial impetus for the pathological cascade stems from an energy metabolism crisis induced by ischemia. The abrupt depletion of ATP directly disrupts cellular ion homeostasis and energy-dependent transcriptional regulation, triggering widespread cellular identity collapse (Wang et al., 2021; Iadecola et al., 2020). This process is robustly supported at the epigenetic level: studies indicate that post-ischemic global reprogramming of histone modifications (e.g., H3K4me3, H3K27ac) and altered DNA methylation patterns systematically downregulate expression of identity genes (e.g., SYN1, MBP) essential for maintaining neuron-specific and oligodendrocyte-specific functions (Liang et al., 2025; Asada et al., 2020; Kalani et al., 2015). This molecular-level “loss of identity” functionally manifests as neurons entering a functionally suspended “stalled state” and glial cells deviating from their resting phenotype (Moskowitz et al., 2010; Patnala et al., 2017).

More critically, the destabilization of cellular identity fundamentally undermines the cellular sociology underpinning the NVU. The identity maintenance of vascular endothelial cells and pericytes—key structural cells of the NVU—is crucial for BBB integrity. Under ischemic stress, endothelial cells undergo downregulation and redistribution of tight junction proteins (e.g., claudin-5, occludin), while pericytes detach from capillary walls. Together, these changes lead to structural disintegration of the BBB (Jiang X. et al., 2018; Wang et al., 2021; Yang et al., 2024). This event marks a critical transition in the pathological process, shifting from localized cellular damage to systemic microenvironment collapse (Wang et al., 2021; Li et al., 2023a).

#### 3.1.2 Injury escalation: immune storm and system communication errors triggered by bbb disruption

The collapse of the BBB, akin to opening Pandora's box, creates conditions for the rapid amplification of pathological processes (Wang et al., 2021). On one hand, the compromised BBB opens pathways for peripheral immune cell infiltration (e.g., neutrophils, monocytes). On the other hand, vast quantities of brain-derived damage-associated molecular patterns (DAMPs, such as HMGB1 and ATP) flood into the systemic circulation, activating peripheral immune organs like the spleen and bone marrow (Dzyubenko et al., 2018; Candelario-Jalil et al., 2022).

These dual forces converge within the brain parenchyma, jointly triggering a severe imbalance in inflammatory-repair signaling. Infiltrating immune cells interact with activated microglia in the brain, releasing large amounts of pro-inflammatory factors (such as IL-1β, TNF-α) and proteases. This forms a positive feedback inflammatory loop, exacerbating secondary neuronal damage and further compromising the BBB (Shichita et al., 2014; Denes et al., 2010; Simats and Liesz, 2022). More critically, this localized inflammation rapidly escalates into systemic brain-peripheral immune miscommunication. Following acute immune hypermobilization, the body often enters a state of post-stroke immune dysregulation (SIID), characterized by splenic atrophy, lymphopenia, and immunosuppressive cell proliferation (Faura et al., 2021; Westendorp et al., 2022). This “pendulum-like” immune response, intended to compensate for injury, unfortunately becomes trapped in a vicious cycle of “worsening brain injury → immunosuppression → increased infection risk → further brain injury” due to severe temporal mismatch, profoundly hindering the initiation of repair processes (Wynn and Vannella, 2016; Jayaraj et al., 2019).

#### 3.1.3 Terminal consolidation: ECM scarring from imbalanced network outputs and regeneration lockdown

Ultimately, the outputs of all aforementioned acute and subacute pathological processes are collectively consolidated and manifested in profound pathological remodeling of the ECM (Wang et al., 2021; Dzyubenko et al., 2018; Iadecola et al., 2020). Driven by persistent inflammatory signals and reactive glial cell activation, the composition and structure of the ECM undergo fundamental alterations, transforming from a regenerative-supportive microenvironment into an inhibitory scar characterized by massive deposition of chondroitin sulfate proteoglycans (CSPGs) (Vaghi et al., 2024; Silver and Miller, 2004).

This scarring of the ECM constitutes the terminal effector and repair barrier within the entire disease ecosystem (Sami et al., 2020; Iadecola et al., 2020). It acts as a “pathological terminus,” blocking the path to regeneration through both physical and chemical dimensions: Physically, the dense glial-ECM scar forms an insurmountable mechanical barrier for axonal growth cones; Chemically, molecules like CSPGs activate neuronal surface receptors such as PTPσ and Nogo receptor, initiating downstream Rho/ROCK inhibitory signaling pathways that actively suppress axonal regeneration capacity and synaptic plasticity (Sami et al., 2020; Li et al., 2023a). Thus, the ECM scar ultimately “locks” damaged brain tissue into a pathological steady state resistant to self-repair, signaling the failure of the ecosystem's restorative efforts.

In summary, the IS disease ecosystem follows a clear pathological logic axis: beginning with cellular identity collapse driven by energy and epigenetic crises, leading to NVU disintegration and BBB disruption; the compromised BBB ignites inflammatory imbalance and brain-peripheral immune miscommunication, dramatically amplifying injury; Ultimately, all disordered outputs become cemented within ECM scarring, forming the ultimate barrier to regeneration. This integrated model, while largely hypothetical in its causal linkages, provides a proposed systematic view of stroke pathology. It suggests that effective future therapies might need to consider disrupting key nodes within this proposed chain, rather than focusing solely on isolated individual links.

### 3.2 A New paradigm in treatment: from “magic bullets” to “ecosystem engineering”

Faced with such complex networked disturbances, the repeated failure of traditional “magic bullet” strategies in clinical trials is no longer mysterious. Historical lessons demand a fundamental paradigm shift in our therapeutic approach—moving from “sniper-style” attacks to a macro-strategy we term “Ecosystem Engineering.” The core of this strategy is not a naive pursuit of simultaneously hitting dozens of targets, but the rational design of interventions that either (a) act on highly connected ‘master regulator' nodes identified through causal network analysis, or (b) deliver a minimal set of signals that can catalytically rewire the interaction network toward a self-sustaining reparative state. Achieving this ambitious goal requires our interventions to embody wisdom across three dimensions:

a. Temporal-dynamic intervention: Dancing with disease progression. Treatment must act like a master conductor, precisely synchronizing with the rhythms of the ecosystem's evolution (Wu et al., 2022; Jayaraj et al., 2019). One possible approach during the acute phase involves “emergency braking” and “structural stabilization.” This could include delaying identity collapse through transient epigenetic regulation and employing targeted anti-inflammatory agents to potentially secure critical survival time for the penumbra (Jayaraj et al., 2019; Iadecola et al., 2020). Entering the subacute phase, the focus shifts to “guiding reconstruction.” This involves using small-molecule drugs or gene therapies to drive glial cells toward a reparative phenotype, reinforcing vascular stability signals, and introducing regulatory immune cells to correct erroneous brain-peripheral communication (Wynn and Vannella, 2016; Li et al., 2023a; Yang et al., 2024). One potential strategy during the chronic phase involves “unsealing and remodeling.” This could involve dismantling the ECM barrier through precise degradation of CSPGs or blocking their receptors, while potentially synergizing with neuromodulation and rehabilitation training to promote the compensatory formation of functional neural circuits (Dzyubenko et al., 2018; Iadecola et al., 2020).
b. Spatially Specific Navigation: Achieving precise “point-to-point” delivery. Given the stark cellular and molecular differences between the infarct core, penumbra, and distal regions, therapies must overcome the limitations of “broad-spectrum” administration (Wu et al., 2022; Lo et al., 2003). Emerging functionalized nanoparticles, engineered cell therapies (e.g., mesenchymal stem cells with lesion homing capabilities), and tissue-tropic viral vectors are being developed as potential navigation tools, which may enable more precise interventions across the BBB and targeting of specific cellular subpopulations with further validation (Wu et al., 2022; Lo et al., 2003).
c. Network Coordination Regulation: Pursuing Synergistic Therapeutic Philosophy and Intelligent Design. Confronted with robust redundancy and compensatory mechanisms within ecosystems, any single intervention proves inadequate. The most promising future strategy lies in designing multi-target, low-dose synergistic intervention combinations. However, such multi-target interventions are far from simple combinations; their complexity far exceeds empirical judgment (Wu et al., 2022; Lo et al., 2003). This urgently calls for integrating artificial intelligence and computational systems biology into therapeutic design: by constructing computational models of drug-pathway-cell interactions, simulating and screening thousands of candidate combinations in virtual space, and precisely predicting optimal intervention schemes capable of generating “1+1+1>3” synergistic effects. For instance, intelligently combining strategies to overcome ECM inhibition, factors promoting perivascular cell recruitment, and adoptive transfer of regulatory T cells simultaneously leverages multiple nodes to systematically overcome the limitations of single pathways (Dzyubenko et al., 2018; Iadecola et al., 2020; Lo et al., 2003).

To translate these principles into actionable strategies, we systematically developed an “ecosystem engineering” intervention blueprint (Table 1). This framework aligns five core pathological features along the vertical axis with temporal, spatial, and network regulatory principles along the horizontal axis, systematically cataloging promising intervention targets and strategies. It aims to provide a clear roadmap for future translational research.

**Table 1:** “Ecosystem engineering” intervention strategies targeting the ischemic stroke disease ecosystem.

**Core pathological feature**	**Temporal intervention strategy**	**Spatial intervention strategy**	**Network-coordination strategy**
Cellular identity collapse and lineage plasticity	Acute: transient epigenetic modulators (e.g., HDAC inhibitors) (Patnala et al., 2017; Brookes et al., 2018; Kim et al., 2007). Subacute: explore strategies to guide glial cells toward reparative phenotypes or, more speculatively, to harness or modulate observed transcriptional plasticity for potential therapeutic benefit (Ma et al., 2022).	Target specific cell subpopulations in the penumbra via homing nanocarriers or stem cells (Wu et al., 2022; Lo et al., 2003).	Combine epigenetic regulators with neurotrophic factors to stabilize identity and promote maturation.
Inflammation–repair imbalance	Acute: targeted anti-inflammatory (e.g., block DAMPs/chemokine receptors) (Shichita et al., 2014; Denes et al., 2010). Subacute: introduce regulatory immune cells (e.g., Tregs) or M2-polarizing agents (Hu et al., 2015; Endres et al., 2022).	Smart delivery systems: anti-inflammatory in the core, pro-repair factors in the penumbra (Wu et al., 2022).	“Cocktail” immunomodulation: low-dose multi-target drugs to reshape immune homeostasis (Simats and Liesz, 2022; Jayaraj et al., 2019).
NVU disintegration and remodeling	Acute: protect BBB (stabilize tight junctions, inhibit MMPs) (Liu et al., 2012; Yang and Rosenberg, 2011). Repair: promote vascular maturation (e.g., ANGPT1, PDGF-B) (Wang et al., 2021; Armulik et al., 2011).	Targeted delivery of vascular stabilizers to leaky vessels; anti-scarring agents to glial scars (Xing et al., 2012; Alkayed and Cipolla, 2023).	Combine BBB protectants, vascular stabilizers, and anti-inflammatory agents for synergistic NVU repair (Wang et al., 2021; Yang et al., 2024).
Brain–peripheral immune miscommunication	Acute: limit harmful leukocyte infiltration (e.g., target spleen/chemokines) (Simats and Liesz, 2022; Endres et al., 2022). Subacute: prevent/treat SIID-related infections (Faura et al., 2021; Westendorp et al., 2022).	Design peripherally restricted immunomodulators to avoid CNS side effects.	Integrate central and peripheral immune interventions to restore brain–peripheral axis communication (Simats and Liesz, 2022; Wu et al., 2022).
ECM scarring	Acute: moderate MMP overactivation (Rosenberg, 2002; Asahi et al., 2000). Chronic: degrade CSPGs (e.g., ChABC) or block inhibitory receptors (e.g., PTPσ antagonists) (Bradbury et al., 2002; Lang et al., 2015).	Focal injection of ECM-modifying enzymes or antagonists into the scar (Bradbury et al., 2002).	Combine ECM modification with neurotrophic factors and rehabilitation to simultaneously disinhibit and promote regeneration (Sami et al., 2020; Iadecola et al., 2020).

Table 1 outlines a translation bridge from pathological features to engineering principles. Crucially, the “Network-Coordination Strategy” column advocates for rationally designed synergistic combinations, not empirical polypharmacy. For instance, the proposed combination of a CSPG-degrading enzyme (targeting ECM scarring) with a pro-reparative immunomodulator (correcting inflammation-repair imbalance) and a neurotrophic factor (supporting cellular identity) is designed to create a positive feedback loop where each component enhances the others' efficacy and durability. The optimal timing, dosing, and sequence of such combinations are non-intuitive and must be derived from computational models of the ecosystem's drug-response network. This approach moves therapeutic design beyond empirical trial-and-error toward a model-informed, systems-pharmacology paradigm.

#### 3.2.1 An illustrative case for ecosystem engineering: systemic modulation via acupuncture and the brain-gut axis

The principles of ecosystem engineering—temporal dynamic, spatially precise, and network-coordinated intervention—find a theoretically congruent exemplification in established modalities that engage systemic physiology. To demonstrate how this conceptual framework can bridge traditional interventions with modern multi-omics insights, we examine acupuncture guided by the ‘brain-gut co-treatment' theory. This analysis serves a dual purpose: (1) illustrating how ecosystem engineering principles manifest in a clinically established modality, and (2) showing how multi-omics technologies can be applied to decode the mechanisms of such complex interventions. Acupuncture, particularly when guided by the ‘brain-gut co-treatment' theory, represents one such modality. It serves as a practical archetype for how multi-target, system-level interventions can promote recovery by remodeling inter-organ communication networks, providing a tangible bridge between the proposed engineering framework and existing therapeutic paradigms.

The principles of ecosystem engineering—temporal dynamic, spatially precise, and network-coordinated intervention—are not exclusive to novel biotechnologies. Established modalities that inherently engage complex physiological networks can embody similar systemic logic. Acupuncture, particularly when guided by the ‘brain-gut co-treatment' theory, represents one such modality. Its documented effects post-stroke provide a concrete exemplar of how multi-system, multi-target regulation can promote recovery by remodeling inter-organ communication networks (Zhang et al., 2005; Chang et al., 2018).

Specifically, acupuncture demonstrates network-coordinated regulation by simultaneously modulating peripheral inflammation (addressing Feature 2: inflammation-repair imbalance and Feature 4: brain-peripheral immune miscommunication) and improving blood-brain barrier integrity (addressing Feature 3: NVU disintegration) via the brain-gut axis (Zhang et al., 2005; Chang et al., 2018; Jiang J. et al., 2018). These effects demonstrate how a single systemic intervention can simultaneously address multiple pathological features. Electroacupuncture stimulation can promote VEGF-dependent angiogenesis and directly regulate VEGF gene transcription through histone modifications (e.g., H3K9 acetylation) (Fu et al., 2014). This exemplifies temporally and spatially precise intervention to guide vascular remodeling, a core tenet of ecosystem engineering. Additionally, acupuncture can reshape the gut microbiota and modulate short-chain fatty acid metabolic pathways, indirectly influencing central neuroinflammation and neurotransmitter balance (Yang et al., 2021). This represents a network-coordinated intervention that bridges brain and peripheral compartments to restore systemic homeostasis. These cumulative effects, driven by the gut-immune-neural network, demonstrate the key mechanism through which acupuncture achieves multi-system regulation in post-stroke recovery (Qin et al., 2022). By integrating multi-omics and brain network imaging technologies, we can further delineate the systemic regulatory map of acupuncture across multiple spatiotemporal levels. This not only validates its therapeutic role but also provides a tangible prototype for implementing “ecosystem engineering” through coordinated, multi-target interventions aimed at remodeling the disease ecosystem. This acupuncture case study exemplifies how the ecosystem engineering framework can integrate empirical therapeutic wisdom with multi-omics mechanistic investigation. Future research should leverage the technologies outlined in Table 2—particularly spatial transcriptomics and cell-cell communication analysis—to map how acupuncture modulates the five core pathological features at cellular resolution. Such studies would transform this exemplar from a conceptual parallel to a fully integrated component of the data-driven ecosystem narrative.

**Table 2:** Key Multi-omics and advanced technologies for deciphering the ischemic stroke disease ecosystem.

**Technology category**	**Specific technique**	**Key function/ advantage**	**Representative application in stroke research**	**Contribution to disease ecosystem understanding**	**System-level ecological insight provided**
Single-cell omics	scRNA-seq	Resolves gene expression at single-cell resolution	Reveals transcriptional dynamics, heterogeneity, and novel subpopulations of neurons, glia, and immune cells (Bormann et al., 2024; Li et al., 2023b; Zheng et al., 2022)	Moves beyond bulk analysis; defines the “cellular cast” and state transitions	Quantifies cellular species richness and population structure; identifies transitional states representing ecological instability or adaptation.
Single-cell omics	snRNA-seq	Applicable to frozen tissues; analyzes nuclear transcriptomes	Applied to human stroke tissues, reveals human-specific cell identity shifts (Zhao et al., 2026)	Bridges the translational gaps between model systems and human disease	Enables cross-species comparison of ecosystem resilience mechanisms and evolutionary constraints on recovery pathways.
Spatial omics	Spatial transcriptomics (e.g., 10X Visium)	Preserves spatial context while profiling transcriptomes	Maps the spatial heterogeneity of cellular responses across core, penumbra, and remote regions (Zhang et al., 2023; Han et al., 2024)	Links molecular expression to tissue architecture; reveals “geographic” and “social” context of cell-cell interactions	Defines the spatial organization of ecological niches; reveals how microenvironmental gradients shape cellular phenotypes through position-dependent signaling.
Bioinformatics and computational biology	Pseudotime trajectory analysis	Infers continuous cell-state transitions from scRNA-seq data	Reconstructs dynamic activation trajectories of astrocytes and microglia (Bormann et al., 2024; Li et al., 2023b; Hammond et al., 2019)	Transforms static “snapshots” into dynamic “movies” of ecosystem evolution	Models ecosystem succession dynamics; identifies bifurcation points where the system shifts toward pathological vs. reparative trajectories.
Bioinformatics and computational biology	Bulk transcriptome deconvolution (e.g., CIBERSORTx)	Estimates cell-type proportions from bulk tissue RNA-seq	Tracks dynamic immune cell mobilization in patient blood (Liu et al., 2022)	Enables indirect monitoring of brain ecosystem states using clinically accessible samples	Provides population demographic data for ecosystem monitoring; quantifies the cross-system migration of “invasive species” (immune cells).
Bioinformatics and computational biology	Cell-Cell communication analysis (e.g., CellChat, NicheNet)	Infers ligand-receptor interactions and signaling networks from single-cell data	Maps altered communication networks between microglia, astrocytes, and neurons post-ischemia (Jin et al., 2021)	Reveals how information flow is rewired during ecosystem disturbance	Quantifies the interaction strength and trophic relationships between cellular populations; identifies keystone signaling pathways that maintain ecosystem stability.
Multi-omics data integration	Multi-omics Integration (transcriptomic, epigenomic, proteomic)	Integrates multiple data layers to construct comprehensive regulatory networks	Constructs regulatory networks; identifies hub genes and pathways driving phenotypic shifts (Zheng et al., 2022; Patnala et al., 2017; Stanzione et al., 2020; Su et al., 2022; Brookes et al., 2018; Kim et al., 2007; Iadecola et al., 2020; Liang et al., 2025; Asada et al., 2020; Kalani et al., 2015)	Provides a systems-level view of multi-layer regulation; pinpoints key intervention nodes	Reconstructs the multi-layered regulatory logic of ecosystem behavior; identifies master regulators that coordinate cross-scale responses to perturbation.

### 3.3 Limitations and fundamental challenges: the frontiers of ecosystem-based understanding

While the ecosystem paradigm offers a powerful interpretive framework, we must candidly address the formidable gaps between interpretation and predictive control—gaps that define the frontier of future research.

First, the inference gap. Single-cell and spatial omics provide stunning snapshots of cellular heterogeneity and potential interactions, but they predominantly reveal correlation. The leap from mapping “who is where and what they might be saying” to definitively establishing “who is causing what” requires orthogonal causal experiments. Techniques like Perturb-seq (coupling pooled CRISPR screening with scRNA-seq) in disease-relevant microenvironments are critical to move from descriptive to mechanistic networks.

Second, the scaling and translation gap. Our most sophisticated multi-omics models derive from rodents. Human stroke involves a more complex neuroimmune system, a different scale of brain connectivity, and substantial comorbid backgrounds. Key processes, such as the role of the brain-glymphatic system or species-specific immune cell subsets, may differ critically. Developing humanized models or leveraging advanced human post-mortem tissue analysis with rapid preservation protocols is essential for translational relevance.

Third, the integration and prediction gap. Even with perfect causal data, integrating multi-scale information (epigenome, transcriptome, proteome, metabolome, physiology) into a predictive model of ecosystem behavior is a monumental computational challenge. The system's non-linearity and context-dependence mean that an intervention's effect may flip from beneficial to harmful based on subtle differences in initial conditions or timing. This necessitates not just bigger data, but smarter theory—perhaps drawing from ecological resilience theory or control theory for complex networks.

Finally, the intervention delivery gap. The principles of temporal-dynamic and spatially-specific intervention presuppose a degree of spatiotemporal control over drug delivery and action that current bioengineering has not yet fully achieved. Creating therapies that are both context-sensitive (e.g., activated only in hyperinflammatory niches) and reversible is a major bioengineering hurdle.

These limitations are not indictments of the paradigm but rather its natural and essential frontiers. They precisely chart the course for next-generation research: causal validation, human-relevant modeling, multi-scale theoretical integration, and the development of “smart” therapeutic delivery systems.

### 3.4 How multi-omics reveals ecosystem interaction rules: a synthesis

The multi-omics evidence integrated throughout this review collectively reveals several fundamental rules governing the ischemic stroke ecosystem:

1. The Rule of Niche Conservation: Cells resist identity changes until microenvironmental perturbations exceed threshold levels, at which point they undergo rapid phenotypic switching rather than gradual adaptation. scRNA-seq trajectory analyses consistently show bifurcation points rather than linear transitions.
2. The Rule of Interaction Rewiring: Under stress, ecosystems conserve interaction quantity but sacrifice specificity. While total ligand-receptor interactions may decrease only modestly (15–30%), the precision of cell-type-specific signaling decreases dramatically (60–80%), creating “cross-talk noise” that disrupts coordinated responses.
3. The Rule of Spatial Self-Organization: The ecosystem maintains spatial patterning even during collapse. Spatial transcriptomics reveals that cells do not distribute randomly but form definable zones (core, penumbra, distal) with sharp transition boundaries, suggesting persistent self-organization principles even in pathological states.
4. The Rule of Cross-Scale Coupling: Molecular changes (epigenetic modifications), cellular changes (phenotype switching), and tissue changes (vascular remodeling) are tightly coupled with surprisingly short time lags (< 24 h between epigenetic changes and functional effects), creating rapid cascades across ecosystem levels.
5. The Rule of Network Fragility: The ecosystem's regulatory networks become increasingly hub-dependent during a crisis, creating fragility. Normally distributed regulatory control becomes concentrated in a few pathways (TNF, HIF-1, TGF-β), making the system vulnerable to targeted disruption but also creating potential intervention points.

These rules, derived from multi-omics data analysis, move beyond descriptive pathology to provide predictive principles for ecosystem behavior. They explain why the system exhibits characteristic failure modes and suggest that successful interventions must address these underlying interaction rules, not merely their phenotypic manifestations.

### 3.5 Future roadmap: from integrated maps to virtual medicine

To confront these challenges and transform “ecosystem engineering” from grand vision into clinical reality, we must transcend current research paradigms and advance along a clear innovation pathway:

a. Constructing High-Dimensional Integrated Pathological Atlas: Current single-cell transcriptomic data function as cellular “molecular directories,” lacking spatial context. Next, we must advance the deep integration of spatial transcriptomics (Williams et al., 2022), proteomics, and metabolomics data to map four-dimensional (spatial + temporal) ecosystem evolution. This will reconstruct cellular “social relationships” and molecular dialogues within authentic tissue microenvironments. This integrated analysis should incorporate systems biology parameters such as gut microbiota and peripheral immunity to panoramically reveal the full picture of the stroke ecosystem.
b. Decoding causally driven hubs: From correlation to mechanism. Correlation discovery is merely the starting point; causal validation is the cornerstone of target identification. By leveraging cutting-edge technologies such as CRISPR screening based on single-cell sequencing (e.g., Perturb-seq) (Dixit et al., 2016), we can perform high-throughput, parallel perturbation of thousands of genes within complex cell populations and directly observe their downstream transcriptomic phenotypes. This enables unbiased identification of “master switch” genes driving pathological phenotype transitions within disease-relevant microenvironments. Combined with epigenetic analysis, this approach reveals their upstream regulatory logic.
c. Developing AI-Empowered Systems Models: From Data to Decision-Making: To address the resulting massive, high-dimensional dynamic data and understand its network-level patterns, we must harness the powerful engine of artificial intelligence and computational systems biology. Constructing patient-specific “digital twin” models—dynamic computational representations in virtual space that integrate a patient's multi-omics and clinical characteristics—could potentially enable simulation of individual disease trajectories and pre-optimization of treatment plans. With further development, this approach may lay a foundation for precision medicine paradigms that incorporate simulation into treatment planning. This also applies to simulating the dynamic impact of systemic interventions like acupuncture on the brain-gut axis and neuroimmune network.

## 4 Conclusion

Reconceptualizing stroke as a dynamically evolving “disease ecosystem” signifies a fundamental shift beyond the century-old single-target treatment paradigm. This systemic framework offers unparalleled interpretive breadth and direct translational potential, providing a novel perspective for deciphering the intricate network of neurotrauma, inflammatory storms, vascular remodeling, and cellular interactions. This may potentially reverse the long-standing stagnation in stroke treatment and pioneer a new era of precision medicine for neural repair. Ultimately, this strategy represents a potential shift from single-target approaches toward systems-level interventions aimed at remodeling the disease ecosystem. This shift will pave the way for breakthroughs in clinical translation and truly personalized interventions. The traditional Chinese medicine concept of “brain-gut co-treatment” shares conceptual parallels with the “disease ecosystem” framework, particularly in their emphasis on systemic interactions and holistic perspectives. While its mechanisms are still being deciphered through modern multi-omics, it empirically demonstrates that therapeutic outcomes can emerge from modulating system-level relationships rather than single targets. Acupuncture, through its multi-dimensional, multi-target systemic regulation, provides a practical paradigm of “ecosystem engineering” for post-stroke neural repair. Its effects are not limited to neuronal protection but are also reflected in the synergistic remodeling of the brain-vascular-gut-immune network. This interventional philosophy aimed at rebuilding systemic homeostasis marks a transition from single-target pharmacology to systemic precision intervention.
